# The effect of Rsk2 on TNFα-mediated bone loss in the TMJ and craniofacial skeleton

**DOI:** 10.1186/s12903-025-05779-9

**Published:** 2025-03-26

**Authors:** Gina Marie Georgi, Frédéric Bachmann, Julia Luther, Anja Derer, Patrick Heimel, Stefan Tangl, Bärbel Kahl-Nieke, Aaron LeBlanc, Jill Helms, Georg Schett, Christian Hirsch, Reinhard Gruber, Michael Amling, Thorsten Schinke, Till Koehne, Julian Petersen

**Affiliations:** 1 Department of Orthodontics, University of Leipzig Medical Center​, Liebigstraße 12, 04103 Leipzig, Germany; 2 Department of Orthodontics, University Medical Center Hamburg-Eppendorf, Hamburg, Germany; 3 Institute of Osteology, and Biomechanics, University Medical Center Hamburg-Eppendorf, Martinistrasse 52, 20246 Hamburg, Germany; 4 Department of Radiation Oncology, Universitätsklinikum Erlangen, Friedrich-Alexander-Universität Erlangen-Nürnberg (FAU), Erlangen, Germany; 5 Department of Oral Surgery, University Clinic of Dentistry, Medical University of Vienna, Vienna, Austria; 6 Austrian Cluster for Tissue Regeneration, Vienna, Austria; 7 Ludwig Boltzmann Institute for Traumatology, the Research Center in Cooperation with AUVA, Vienna, Austria; 8 Centre for Oral, Clinical & Translational Sciences, Faculty of Dentistry, Oral & Craniofacial Sciences, King’s College London, London, UK; 9 Department of Surgery, Stanford University School of Medicine, Stanford, CA USA; 10 Deutsches Zentrum Für Immuntherapie (DZI), Friedrich Alexander University Erlangen-Nuremberg, and Universitätsklinikum Erlangen, Erlangen, Germany; 11 Department of Internal Medicine 3-Rheumatology, and Immunology, Friedrich Alexander University Erlangen-Nuremberg, and Universitätsklinikum Erlangen, Erlangen, Germany; 12 Clinic of Pediatric and Preventive Dentistry, University of Leipzig, Liebigstr. 12, 04103 Leipzig, Germany; 13 Department of Oral Biology, University Clinic of Dentistry, Medical University of Vienna, Vienna, Austria

**Keywords:** Geometric morphometrics, Inflammation, Rheumatoid arthritis, Transgenic mouse, Temporomandibular joints, Tumor necrosis factor-α, ribosomal s6 kinase 2

## Abstract

**Objectives:**

This study aims to investigate the impact of the pro-osteoblastogenic ERK-activated ribosomal S6 kinase (*Rsk2)* on Tumor necrosis factor (TNF)α-induced bone loss in the craniofacial system, focusing on its role in rheumatoid arthritis (RA). The objective is to understand whether *Rsk2*, previously shown to have protective effects in long bones against TNFα-induced bone resorption, exhibits similar effects in the craniofacial region.

**Materials:**

, and Methods.

The study compares mice with TNFα overexpression, *Rsk2* knockout mice, and a combination of TNFα, and *Rsk2* knockout mice using detailed micro-computed tomography coupled with landmark based morphometric analysis, and classical histology. The overall skull morphology, mandible shape, and the temporomandibular joint were examined. Additionally, histological sections were utilized to examine the synovial membrane.

**Results:**

Combining TNFα, and *Rsk2* deficiency does not further alter overall skull shape compared to TNFα alone. TNFα overexpression shortens the mandibular ramus, exacerbated by *Rsk2* absence. Micro-computed tomography (µCT) reveals significant temporomandibular joint damage from TNFα, independent of *Rsk2*. However, histological sections show increased synovial membrane thickness with TNFα, heightened in the absence of *Rsk2*.

**Conclusions:**

*Rsk2* mitigates TNFα-induced effects on mandibular ramus length in the craniofacial system but has limited impact on the temporomandibular joint, except for synovial membrane thickness. Overall, *Rsk2* demonstrates a weaker preventive effect on TNFα-induced craniofacial bone loss compared to its established role in the appendicular skeleton.

**Clinical Relevance:**

This study highlights regional differences in *Rsk2's* protective mechanisms, emphasizing the need for further exploration of the underlying mechanisms for these disparities. Understanding these regional differences can be crucial for the development of targeted therapeutic interventions.

**Supplementary Information:**

The online version contains supplementary material available at 10.1186/s12903-025-05779-9.

## Introduction

Bone remodeling is a dynamic, and lifelong process involving a finely tuned balance between bone formation, and resorption, ensuring skeletal homeostasis [1]. The process is tightly regulated, and coordinated by various cell types, with osteocytes, osteoblasts, and osteoclasts being the primary cells involved in bone formation, and resorption [2].

Disruption of this balance leads to bone pathologies such as osteoporosis, and inflammatory bone loss. One of the most frequent pathological settings causing deregulated bone remodeling is chronic inflammation [3]. In this study, a murine model of systemic TNFα overexpression was employed to mimic the inflammatory environment observed in RA. This model allows for detailed analysis of the effects of cytokine-driven inflammation on bone and joint health, specifically focusing on the craniofacial system. The cytokine-triggered inflammatory cascade within the synovium has a significant impact on this process. In murine models, systemic overexpression of Tumor necrosis factor α (TNFα) causes complex deregulation of bone metabolism [4], and TNFα blockade therapy in humans has been shown to play a role in the pathogenesis of inflammatory bone resorption through inflammation [3].

The impact of TNFα is complex, but can negatively impact bone homeostasis through increased osteoclast proliferation, reduced osteoblastogenesis, as well as induction of osteoblast apoptosis by triggering Runx2, and Osterix [5, 6].

Interestingly, in patients with rheumatoid arthritis (RA), the S6 kinase RSK2 was found to be highly activated in joints, providing protection against inflammation, and increased destruction of bone, and cartilage [7]. This kinase, is a downstream effector of the Ras–extracellular signal-regulated kinase (ERK)/mitogen-activated protein kinase (MAPK) signalling cascade [8], which in turn is regulating the transcription factors AP-1, which plays a significant role in bone remodeling [9, 10].

In addition to this, RSK2 inhibits TNFα-induced apoptosis of osteoblasts through the activation of the Nuclear factor kappa-light-chain-enhancer of activated B cells (NF-kB) pathway [11]. Mice overexpressing TNFα in combination with inactivated RSK2 exhibited reduced bone mass, decreased osteoblast, and increased osteoclast counts, as well as elevated expression of osteoblast, and osteocyte markers in their long bones [4]. However, whether this holds true for the craniofacial system has remained unexplored until now. Whether this phenomenon is exclusive to long bones or is also applicable to craniofacial bones remains an important question, as the prevalence of RA is increasing among patients [12].

RA frequently results in chronic pain, and joint dysfunction including limitations in joint mobility, and crepitus [13]. This can lead to painful swelling, reduced biting force, and restricted mouth opening [14]. RA is a multi-factorial disease with a complex pathogenesis involving not only inflammation, but angiogenesis, innervation, and in some cases ossification [15, 16]. RA is an autoimmune disorder characterized by systemic inflammation, where pro-inflammatory cytokines, particularly TNF-α, play a significant role. TNFα acts as a key regulator by modulating the production of various other pro-inflammatory cytokines [17]. Upon experiencing stress, infection, or injury, macrophages, synoviocytes, and neurons connected to the trigeminal ganglion release TNFα, which results in myofascial pain, and temporomandibular joint (TMJ) inflammation [18]. Subsequently, TNFα's impact on cells, specifically chondrocytes, and synovial fibroblasts, causes pathological degradation of cartilage in the temporomandibular joint [17]. Furthermore, various downstream cytokines, and neuropeptides influenced by TNFα can regulate central sensitization, and inflammatory pain in Temporomandibular disorders (TMD) [17]. Therapeutic strategies are usually symptomatic, and none can cure TMD [13].

Therefore, this study aimed to investigate whether RSK2 can also protect craniofacial bones against TNFα-induced bone loss in mice, using a TNFα overexpression model. By linking the established effects of TNFα in systemic bone pathology with the less-studied implications for TMJ health, we hope to provide new insights into the management of RA-related TMJ disorders.

## Material, and methods

### Laboratory animals

All mice were maintained in a C57/Bl6 background in the animal facility of the University of Erlangen-Nuremberg. Our study did not involve a clinical trial registration number. The animal experiments presented in this manuscript were approved by the local ethic committee of the Regierung von Mittelfranken, Germany. This approval did not involve interventions that required clinical trial registration. Animal care, and handling as well as all experimental procedures were performed in accordance with all national, and european guidelines. All methods were performed in accordance with the relevant guidelines, and regulations. The study is reported in accordance with ARRIVE guidelines. Animals were euthanized by CO2 inhalation. The heads were fixed overnight in 4% formalin, and then transferred to 70% ethanol. We have further described the methods for producing, and handling the mice in a prior publication [4]. In this study, the skulls of four mice per group were investigated to further characterize the effect of RSK2 on the craniofacial system. Four mouse strains are involved. These are *hTNFα-tg* (heterozygous transgenic mice for human TNF, previously described here [19], *Rsk2*^*−/y*^ (Rsk2-deficient mice), *hTNFα-tg*;*Rsk2*^*−/y*^ (Rsk2-deficient in combination with *hTNFα-tg* mice), and the corresponding control litter mate mice. Since *Rsk2* is inherited X-linked, only male animals were used for the study. The animals were examined at 10 weeks of age. We chose to conduct testing at 10 weeks of age because, in our previous study on long bones, we found that while wild-type, and Rsk2^−/y^ mice did not experience mortality, all hTNFα-tg mice succumbed before reaching 17 weeks, and hTNFα-tg;Rsk2^−/y^ mice did not survive beyond 11 weeks. To ensure a sufficient number of living hTNFα-tg;Rsk2^−/y^ mice for comparison, we selected 10 weeks as the optimal age for our study.

In total, the skulls of four animals of each of the four different genotypes could thus be analyzed. All animal experiments were performed with the approval of the local ethics committee. The study is reported in accordance with ARRIVE guidelines.

### µCT

Micro-computed tomography (µCT-40, SCANCO Medical, Brüttisellen, Switzerland) was used to scan the skulls of the experimental animals. Scans were performed with 55 kVp, 145 µA. 1000 projections per 180° were integrated for 200 ms, and reconstructed to an isotropic resolution of 15 µm. Three-dimensional analysis was performed using the integrated software. Avizo 3D-Pro (version 2022.2-Thermo Fischer Scientific Inc., Waltham, United States) was used for semi-automatic segmentation of the skull, and mandible, and for the measurements of volume, and linear distances (**Supplementary Fig. 1**). For a better visualization a wall-thickness analysis was performed on each specimen.

### Temporomandibular joint by µCT

A Definiens rule set was created to quantify the bone erosion in µm, and the superficial bone volume/tissue volume in (%) for the mandibular condyle, and the articular fossa [20]. Erosion was measured as the average distance of a computed smoothened surface to the real surface. Bone volume, and tissue volume were determined within a 150 µm zone from the smoothed bone surface. The outer edge of the bone was smoothed using surface tension constrained region growing.

### Histology

Fixed skulls were halved along the median plane before one of the halves underwent a 14-day decalcification process in a solution bath (Medite GmbH USEDECALC). To maintain the frontal plane during sectioning on the microtome, the skulls were trimmed orthogonal to their longitudinal axis behind the auditory canal. The specimens were dehydrated overnight, and subsequently embedded in 60 °C warm paraffin the next day. Using a Leica RM2245 microtome (Leica Biosystems GmbH), the temporomandibular joint was sectioned at a thickness of 3 μm, covering the mesio-distal area, under microscopic control. The sections were transferred using a transfer basin (pfm Waterbath 1000 49 °C, pfm medical ag) onto slides (polysine-coated, Fa.Menzel GmbH) before being fixed in a heating cabinet at 37 °C for one hour. Hematoxylin, and Eosin (H&E) staining, and toluidine blue staining were performed following the standardized protocols. The sections that were stained were rinsed with distilled water, and dehydrated in a series of ascending alcohols. Prior to being covered with Eukitt (ORSAtec GmbH, Bobingen, Germany), the sections underwent infiltration with xylene in three successive baths for 5 min each.

The histological evaluation of the TMJ for cartilage morphology changes was performed using the OsteoArthritis Research Society International (OARSI) grading system. The OARSI mouse OA semi-quantitative grading system has 6 grades corresponding to the degree of cartilage loss: Grade 1—no cartilage loss, but reduced safranin-O staining; Grade 2—some superficial vertical clefts of the cartilage with some loss of the surface lamina; Grade 3—cartilage destruction of less than ¼ of its thickness; Grade 4—cartilage destruction reaching ½ of its thickness; Grade 5—cartilage destruction of ¾ of its thickness; Grade 6—subchondral bone denudation, so that more than ¾ of the cartilage is destroyed [21].

### Morphometric analysis

Geometric morphometrics is a quantitative method used in biological research to analyze, and compare the shape, and size of biological structures, such as bones or organisms, using geometric coordinates. It employs mathematical, and statistical techniques to extract, and analyze shape variation. Here, geometric morphometrics of the skull have already been used to study skull shape change in genetically modified mice [20, 22]. Skulls were visualized in Avizo 3D-Pro (version 2022.2-Thermo Fischer Scientific Inc., Waltham, United States) as an isosurface. Isosurface threshold levels were selected manually. For placing landmarks in 3D, the inbuilt landmark editor of Amira was used. A single person placed all landmarks. No semilandmarks were used. Procrustes superimposition, and principal component analysis were conducted using MorphoJ [23]. For visualization purposes 3D landmarks were manually connected with lines in MorphoJ.

### Statistical evaluation

For all analysis, each measurement was performed blind, i.e., only the supervisor knew the assignment of the individual samples. Graphs were plotted, and statistical analysis of the data was performed using GraphPad PRISM 8 software (GraphPad Software Inc., San Diego, California, United States). To focus on the essentials, and not overcrowding the graphs we decided to concentrate our analysis on the following groups i) control vs. *Rsk2*^*−/y*^ ii) control vs*. hTNFα-tg* iii) control vs. *hTNFα-tg;Rsk2*^*−/y*^*, and* iv) *hTNFα-tg* vs. *hTNFα-tg;Rsk2*^*−/*y^. The one-way ANOVA was used to assess whether there were statistically significant differences in the mean values across the groups. Post-hoc analysis was performed to identify specific group differences, and multiple comparisons were corrected using the Šidák correction. This correction controlled the family-wise error rate to maintain a 95% confidence level. The Šidák correction ensures that the probability of making one or more type I errors across all comparisons remains below the predetermined family-wise alpha threshold, providing robust results in our hypothesis testing. The significance value or p-value was marked with asterisks as follows: p ≤ 0.05 = *, p ≤ 0.01 = **, and p ≤ 0.001 = ***.

The sample size which we used in the study was based on the following considerations: Feasibility: Due to constraints in animal availability, and ethical guidelines, we utilized the maximal number of samples feasible. This in particular due to the fact that hTNFα-tg;Rsk2-/y mice have a very severe phenotype, and do not lived past 11 weeks.

Validation, and Robustness: The results have proven consistent across different methods, and comparisons, reinforcing the reliability of our findings.

Taking these considerations into account, we performed a sample size calculation using STATA (version 15). For this purpose, we assumed a 0.5 mm difference in the measurements and an accuracy (standard deviation) of 0.25 mm in accordance with a CBCT/Micro-CT comparison study by [24]. This results in a sample size of 4 test animals each.

## Results

### Rsk2 deficiency does not alter TNFα-induced changes in the skull

**Fig. 1:**
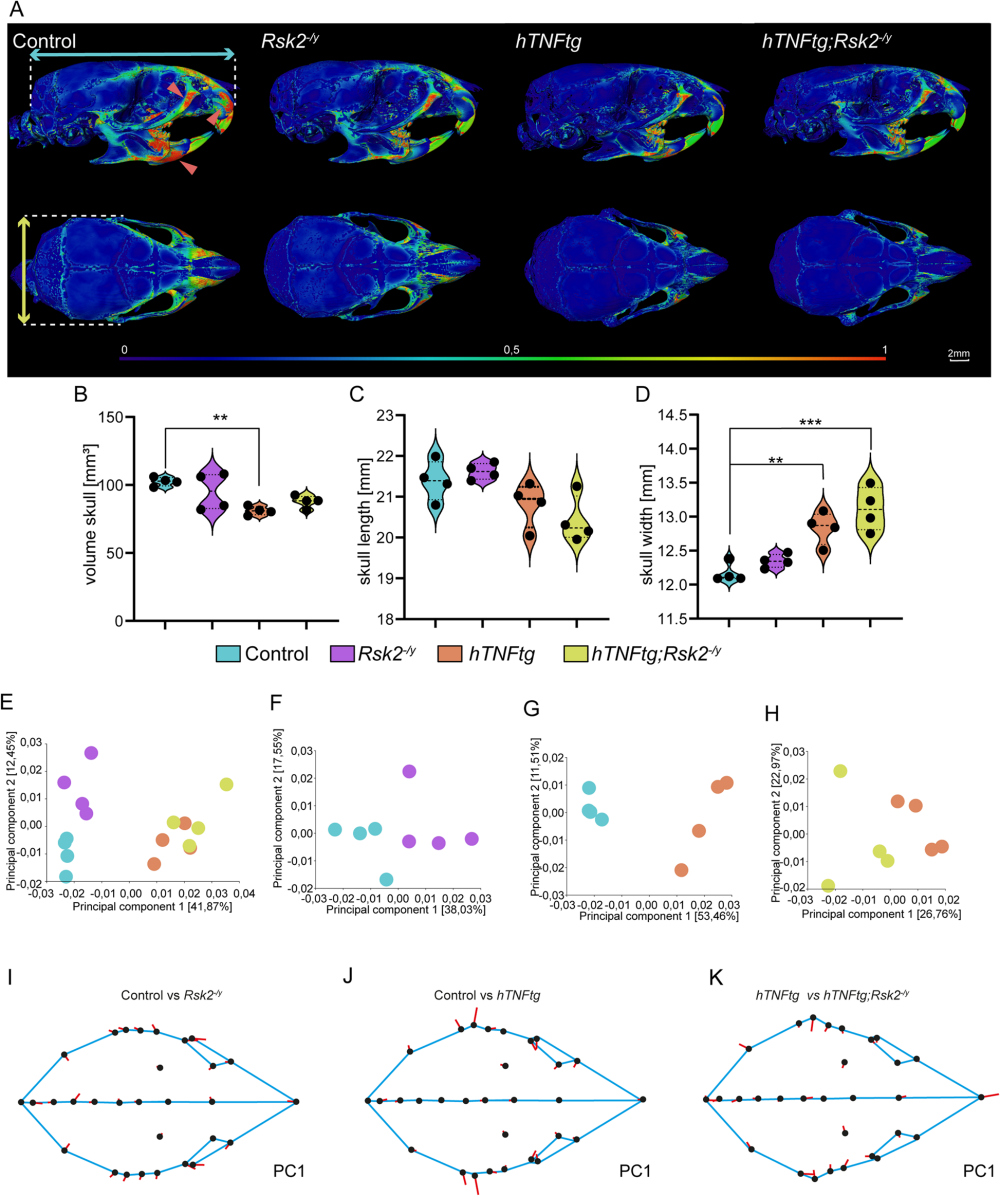
Craniofacial Skeleton Analysis in Mice with RSK2, and TNFα Genetic Modifications. **A** Comprehensive evaluation of craniofacial morphology in 10-week-old mice through µCT scans. The study groups include control mice, RSK2 knockout (*Rsk2*^*−/y*^), TNFα overexpressed (*hTNFα-tg*) mice, and a combination of TNFα overexpression, and RSK2 knockout (*hTNFα-tg;Rsk2*^*−/y*^). The upper panel displays left lateral views, while the lower panel presents a superior view of the skulls. **B** Quantitative assessment of total skull volume. **C** Precise measurements of skull length (indicated by the blue arrow). **D** Precise measurements of skull width (indicated by the yellow arrow). Scale bar: 2 mm, **p < 0.01, ***p < 0.001. **E**–**H** Principal Component Analysis of the geometric morphometric approach along the Principal Components 1, and 2 (PC). **I-K**: Geometric morphometric analysis on mean group shape visualized as Lollipop graphs according to PC1. Landmarks were connected with lines to mimic skull shape. *N* = 4 for all groups

To investigate the potential involvement of *Rsk2* in mediating the effects of TNFα on the craniofacial skeleton, we analysed a mouse model with TNFα overexpression, and a genetic deficiency of *Rsk2*. This model, referred to as *hTNFα-tg;Rsk2*^*−/y*^, was generated through the crossbreeding of *hTNFα-tg* mice [19] with *Rsk2* knockout mice [25, 26]. We first used high resolution µCT followed by detailed Amira Avizo analysis to examine 10 week old skulls of *hTNFα-tg*, *Rsk2*^*−/y*^, and *hTNFα-tg*;*Rsk2*^*−/y*^ in comparison with littermate WT controls (Fig. 1).

We observed a reduction in bone thickness in specific craniofacial regions, specifically the zygomatic, mandibular, and maxillary areas in *Rsk2*^*−/y*^, *hTNFα-tg*, and *hTNFα-tg;Rsk2*^*−/y*^ mice compared to controls (Fig. 1A highlighted by red arrow).

A more detailed analysis of the volume, length, and width of the skulls revealed small but subtle changes. In particular, changes in skull volume, and width were evident in *hTNFα-tg* mice, with only a slight, and non-significant increase in the absence of *Rsk2*. (Fig. 1B-D**)**. In contrast, the cranial morphology of the *Rsk2*^*−/y*^ group remained largely unaffected (Fig. 1B-D**)**. Furthermore, the skull length across the different groups remained unaffected with a slight but non-significant decrease evident in the *hTNFα-tg*, and *hTNFα-tg;Rsk2*^*−/y*^ groups (Fig. 1C).

Next, we performed landmark-based geometric morphometric analysis using 30 defined landmarks across the skull (Fig. 1E-H**, **and Supplementary Fig. 3). At first, we compared all four groups using principal component analysis (PCA), revealing morphological differences in all groups with Principal component 1 (PC1) explaining 41.87%, and Principal component 1 (PC2) explaining 12.45% of the variation (Fig. 1E**)**. To explore the specific differences between the groups, we compared the groups separately. Here control, and *Rsk2*^*−/y*^ skulls revealed morphological differences, with PC1 explaining 38.03%, and PC2 explaining 17.55% of the variation (Fig. 1F**)**. The morphometric analysis of control, and *hTNFα-tg* skulls demonstrated a pronounced divergence. PC1 accounted for 53.46%, and PC2 for 11.51% of the variation (Fig. 1G**)**. The analysis of *hTNFα-tg*, and *hTNFα-tg;Rsk2*^*−/y*^ skulls revealed that PC1 accounted for 26.76% of the variation, while PC2 explained 22.97% (Fig. 1H**)**.

To visually elucidate the underlying variations, lollipop graphs were employed (F ig. 1I-K**)**. Analyzing these lollipop graphs allows for a more detailed exploration of the morphological distinctions, aiding in the identification of key characteristics that contribute to the observed variations. Here we show the differences along PC1 for control vs *Rsk2*^*−/y*^ (F ig. 1I)**,** control vs *hTNFα-tg* (Fig. 1J**), and** *hTNF* vs *hTNFα-tg*;*Rsk2*^*−/y*^ (Fig. 1K**).** In particular, the lollipop graph highlighted an enlargement of the zygomatic process in the *hTNFα-tg* compared to the control group (Fig. 1J**)**.

**Fig. 2:**
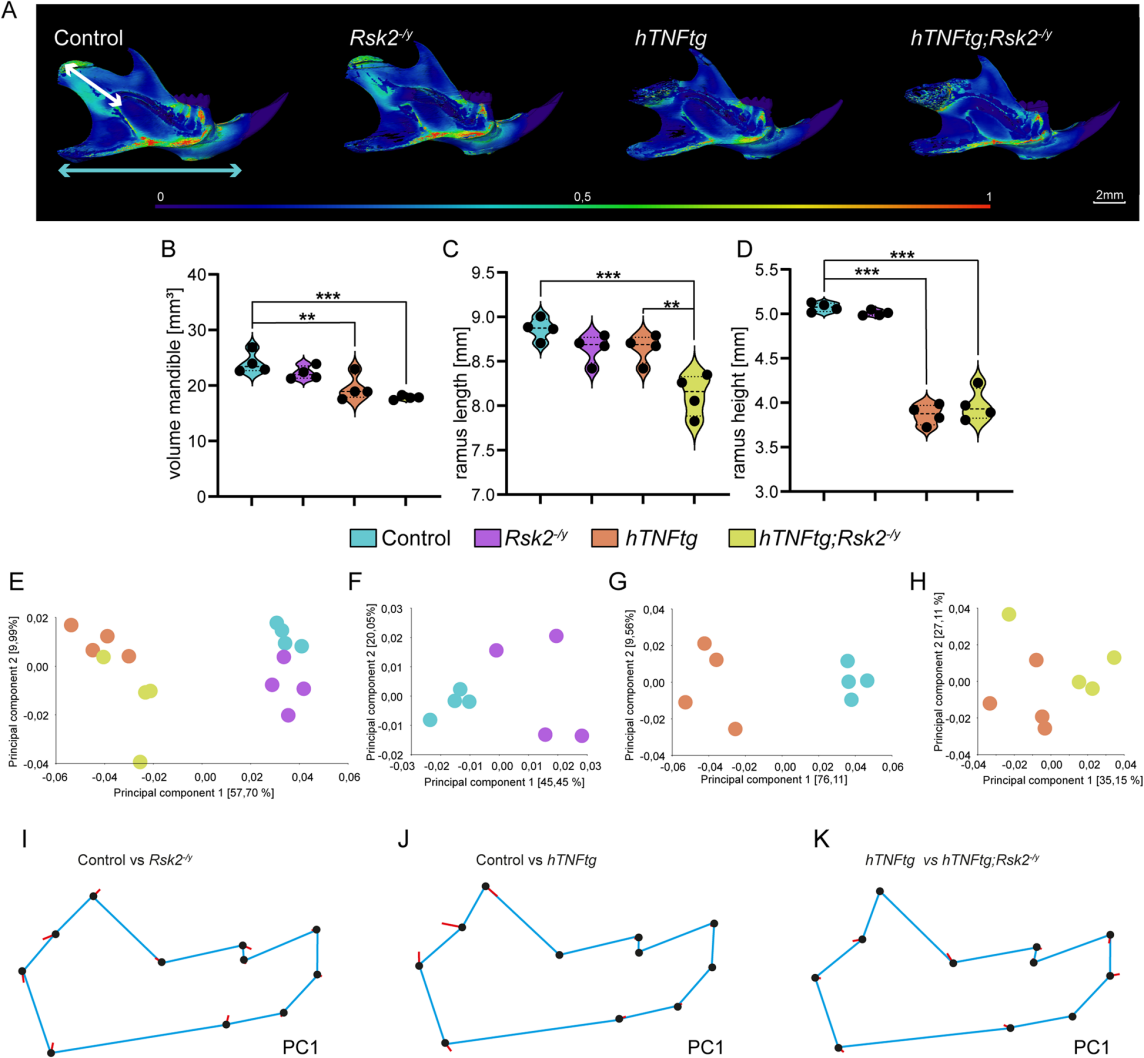
Mandibular Analysis in Mice with RSK2 Overexpression, and TNFα Knockout. **A** Thorough examination of mandibular bone characteristics through µCT scans in 10-week-old mice. The study groups consist of control mice, RSK2 knockout (*Rsk2*^*−/y*^), TNFα overexpressed (*hTNFα-tg*) mice, and a combination of TNFα overexpression, and RSK2 knockout (*hTNFα-tg; Rsk2*^*−/y*^). The figure offers a lateral view of the mandibles. ) Quantitative assessment of mandible volume. **C** Precise measurements of ramus length (indicated by the white arrow). **D** Precise measurements of ramus hight (indicated by the cyan arrow). Scale bar: 2 mm, **p < 0.01, ***p < 0.001. **E**–**H** Principal Component Analysis of the geometric morphometric approach along the Principal Components 1, and 2 (PC). **I-K** Geometric morphometric analysis on mean group shape visualized as Lollipop graphs according to PC1. Landmarks were connected with lines to mimic mandible shape. *N* = 4 for all groups

Next, we concentrated on the mandible, where we observed significant changes in both shape, and wall thickness resulting from TNFα overexpression (both on the *hTNFα-tg*, and *hTNFα-tg;Rsk2*^*−/y*^ group compared to controls) (Fig. 2A). *Rsk2*^*−/y*^ mice on the other hand were not visibly affected. A more detailed quantification of the volume, ramus length, and ramus height further revealed the drastic differences between the TNFα overexpression groups, and controls (Fig. 2B-D). Interestingly, the overexpression of TNFα led to a reduction in the length of the ramus mandibulae, and this effect became more pronounced in the absence of *Rsk2* (Fig. 2C).

Following the initial examination of mandible characteristics, we performed next landmark-based geometric morphometric analysis using 11 defined landmarks across the mandible (Fig. 2E-H**,** and Supplementary Fig. 3). The principal component analysis revealed significant distinctions among the groups, with PC1, and PC2 serving as key parameters for comparison. For the mandibular analysis, when comparing all groups, PC1 accounted for 57.70% of the variation, while PC2 values were 9.99% (Fig. 2E). Notably, the comparison between the control, and *Rsk2*^*−/y*^ groups exhibited PC1, and PC2 values of 45.45%, and 20.05%, respectively (Fig. 2F). Further comparisons included control vs *hTNF*, showcasing PC1, and PC2 values of 76.11%, and 9.56% (Fig. 2G), and *hTNF* vs *hTNF*;*Rsk2*^*−/y*^, where PC1, and PC2 values were observed as 35.15%, and 27.11% (Fig. 2H).

Next, we employed lollipop graphs to visually elucidate the underlying variations (Fig. 2I-K**)**. Here we show the differences along PC1 for control vs *Rsk2*^*−/y*^ (Fig. 2I)**,** control vs *hTNFα-tg* (Fig. 2J**), and** *hTNFα-tg* vs *hTNFα-tg*;*Rsk2*^*−/y*^ (Fig. 2K**).** In particular, the lollipop graph highlighted the strongest variations within the zygomatic process in the *hTNFα-tg* compared to the control group (Fig. 2J**)**.

### Rsk2 deficiency has little impact on TNFα-induced condylar bone degeneration in the TMJ

**Fig. 3:**
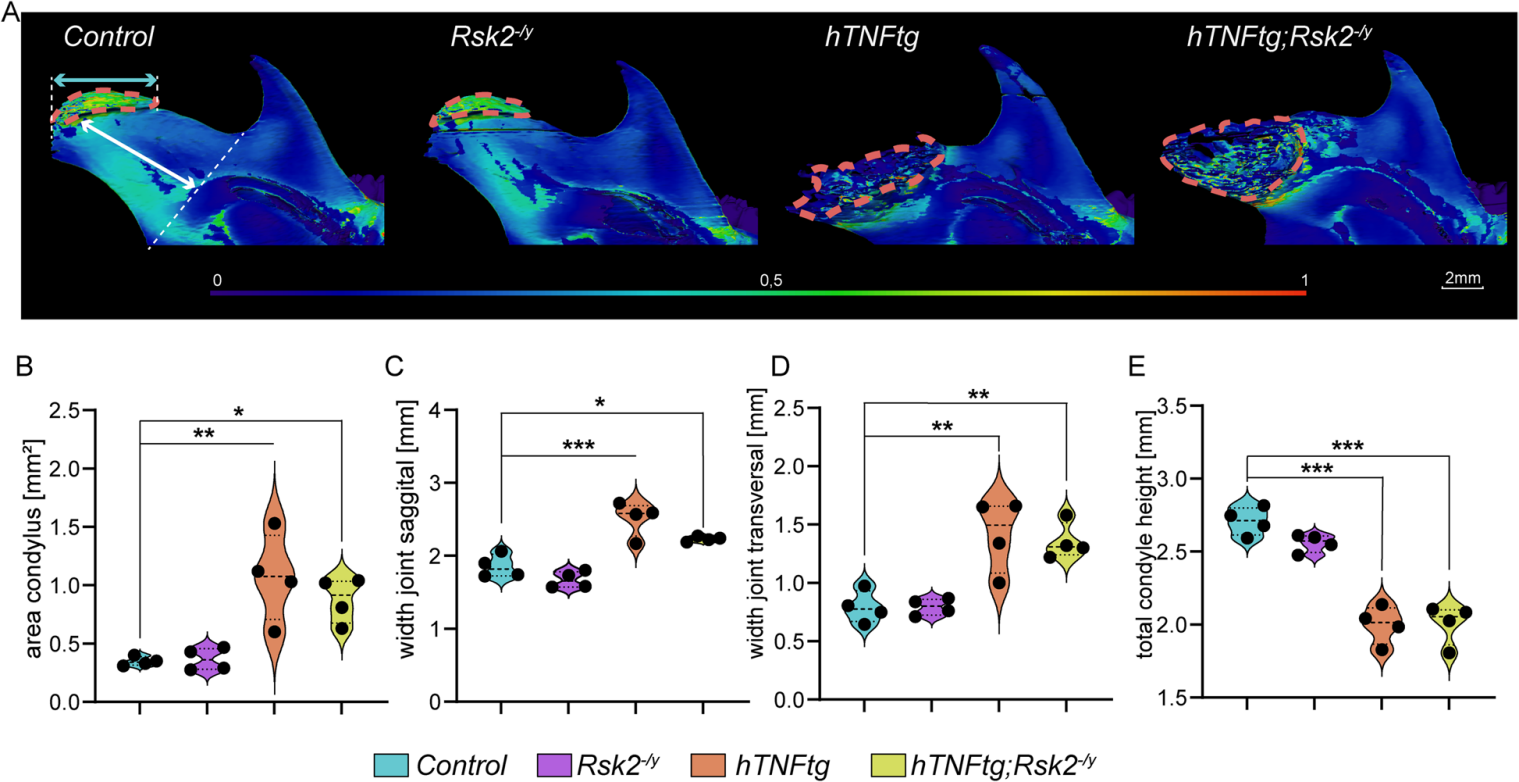
Condyle Analysis in hTNFα-tg, and hTNFα-tg;Rsk2^−/y^ Mice. **A** Thorough examination of the condyle through µCT scans in 10-week-old mice. The study groups consist of control mice, RSK2 knockout (*Rsk2*^*−/y*^), TNFα overexpressed (*hTNFα-tg*) mice, and a combination of TNFα overexpression, and RSK2 knockout (*hTNFα-tg*; *Rsk2*^*−/y*^). **B** Quantitative assessment of the condyle area (**C**) Precise measurements of sagital joint width (indicated by the cyan arrow). **D** Precise measurements of transversal joint width. **E** Precise measurements of the total condyle height (indicated by the white arrow). Scale bar: 2 mm, *p < 0.05, **p < 0.01, ***p < 0.001. *N* = 4 for all groups

Since TNFα overexpression is known to heavily effect joints, and cartilage [27], we next focused on the condylar bone, and the temporomandibular joint (Fig. 3). Here, overexpression of TNFα resulted in a larger, and deteriorated condyle while deletion of *Rsk2* did not lead to overall changes in the condyle (Fig. 3A). Moreover, detailed analysis further revealed significant changes associated with overexpression of TNFα (*hTNFα-tg*, and *hTNFα-tg;Rsk2*^*−/y*^) on the condylar surface area (Fig. 3B**),** sagittal joint width **(**Fig. 3C**)** transversal joint width **(**Fig. 3D**), **and total condyle height **(**Fig. 3E**).** Interestingly, the absence of *Rsk2* did not pronounce these changes (Fig. 3B-E**)**.

**Fig. 4:**
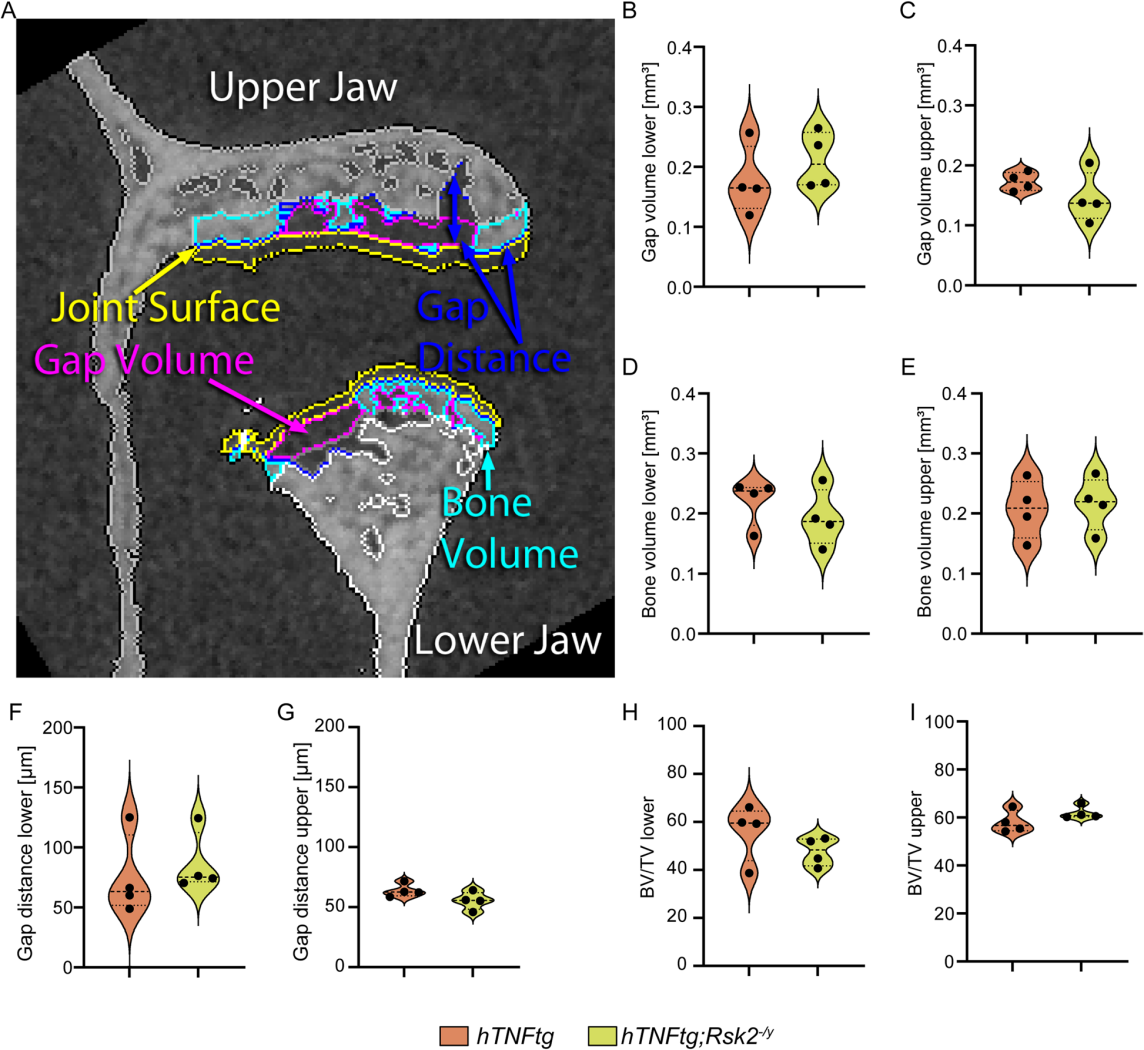
Detailed µCT Examination of the Temporomandibular Joint (TMJ) in hTNFα-tg, and hTNFα-tg;Rsk2^***−/y***^ Mice. **A** Cross-sectional view of the TMJ, encompassing both the upper, and lower jaws. Key measurements in the top 150 um of the joint surface include gap distance (blue), gap volume (purple), joint surface (yellow), and bone volume (turquoise). Quantification (separately for the upper, and lower jaws) of: (**B**, **C**) Gap volume. **D**, **E** Bone volume. **F**, **G** Gap distance. **H**, **I** Bone volume fraction (BV/TV). *N* = 4 for all groups

Given that these data contrast with current research on other joints, where Rsk2 has been shown to exhibit a protective effect on long bones [4] we conducted a more detailed analysis comparing *hTNFα-tg*, and *hTNFα-tg;Rsk2*^*−/y*^ mice in a 150 µm wide region using Definiens Developer (Fig. 4**,** and Supplementary Fig. 2). Here we separately analyzed the lower, and upper condyle as well as gap distance, gap volume, joint surface, and bone volume (Fig. 4A). Interestingly, while none of these parameters showed significant differences between the two groups, this finding is noteworthy as it suggests that the protective effects of Rsk2 observed in long bones may not extend to the craniofacial region when TNFα is overexpressed in combination with *Rsk2*^*−/y*^ (Fig. 4 B-I). This lack of significant change highlights the need for further investigation into the role of Rsk2 in different bone types, and joints, as its effects may vary depending on anatomical, and pathological contexts.

**Fig. 5:**
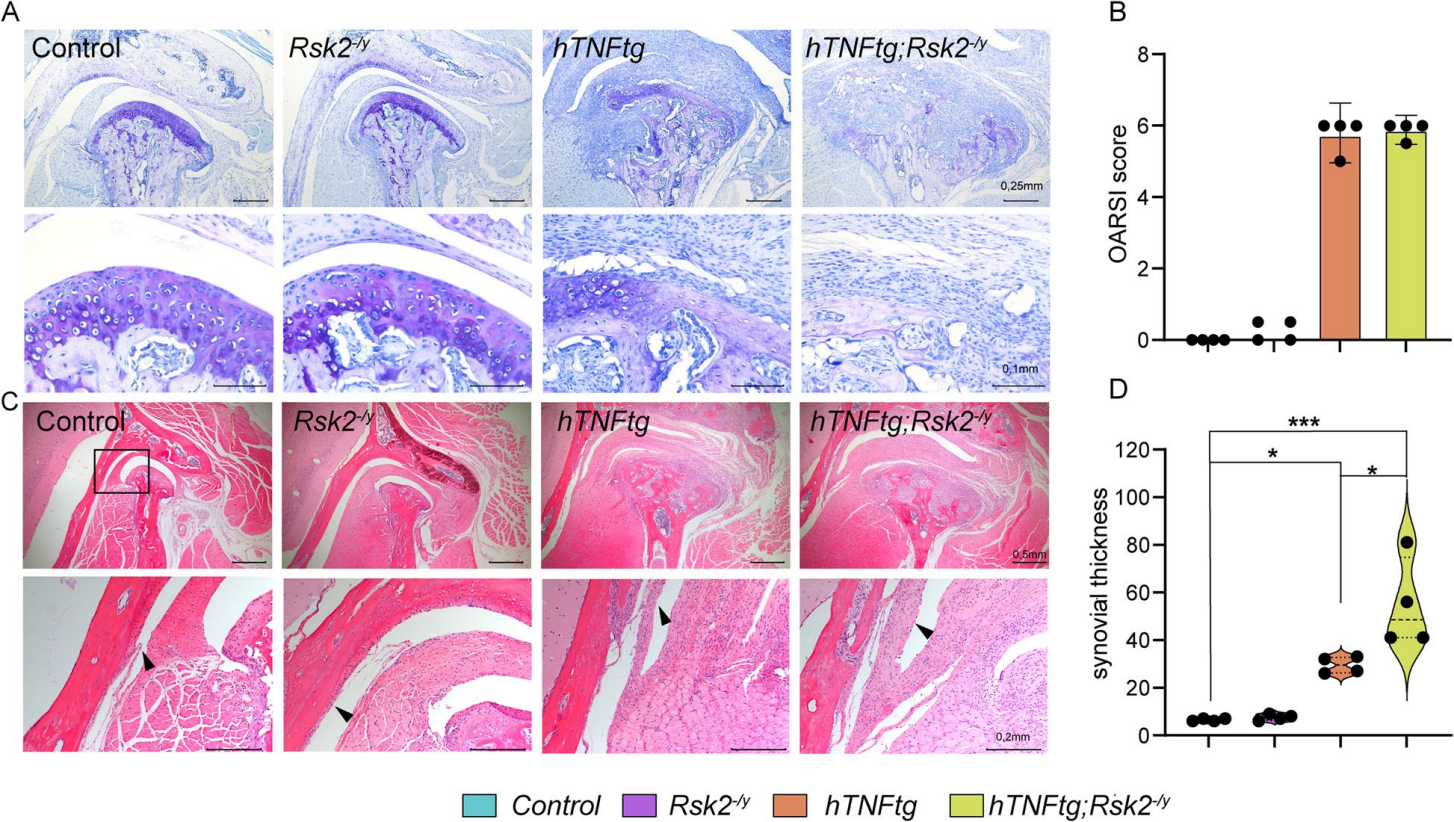
Histological Evaluation of the Temporomandibular Joint (TMJ) in 10-Week-Old Mice with Various Genetic Modifications. **A** Utilization of Toluidine Blue staining to visualize OARSI-Score. The upper panel provides an overview, while the lower panel offers a close-up view of the cartilage. This staining method was employed for quantifying osteoarthritic damage (**B**), graded as follows: 0 = Normal; 0.5 = Loss of Safranin-O without structural changes; 1 = Small fibrillations without loss of cartilage; 2 = Vertical clefts down to the layer immediately below the superficial layer, and some loss of the surface lamina; 3 = Vertical clefts/erosion to the calcified cartilage extending to < 25% of the articular surface; 4 = Vertical clefts/erosion to the calcified cartilage extending to 25–50% of the articular surface; 5 = Vertical clefts/erosion to the calcified cartilage extending to 50–75% of the articular surface; 6 = Vertical clefts/erosion to the calcified cartilage extending > 75% of the articular surface. **C** Hematoxylin–Eosin staining to visualize synovial thickness. The upper panel displays an overview, while the lower panel offers a close-up view of the synovial membrane. The thickness of the synovial membrane was quantified (**D**). *p < 0.05, ***p < 0.001

Lastly, we performed histological sections of the condyle, and the temporomandibular joint (Fig. 5). Here, we first quantified changes using an established mouse score from the OARSI. All controls as well as *Rsk2*^*−/y*^ mice exhibited an intact cartilage layer (score 1), whereas the TNFα-overexpressing mice showed an advanced stage of arthritis with bony erosions, and erosions, and cartilage loss of more than 50% (score 5), and 75% (score 6) (Fig. 5C). Interestingly, the combination of TNFα overexpression, and the knockout of *Rsk2* did not further change the OARSI score. However, the thickness of the synovial membrane significantly increased (Fig. 5D).

In summary, our results suggest that *Rsk2* has a minor protective role against TNFα-mediated bone loss in the craniofacial region.

## Discussion

Despite being one of the most common joints affected by arthritis, the temporomandibular joint receives minimal attention in arthritis research. To remedy this the aim of this study was to investigate the protective effect of the ribosomal S6 kinase Rsk2, which is known to act downstream of the inflammation cascade induced by TNFα [11]. Using a mouse model overexpressing TNFα we identified significant impacts on the development of craniofacial skeleton as well as the rheumatoid arthritis (RA)-affected joints, and the ascending ramus. Geometric analysis revealed significant changes, including a reduction in the lower jaw height, shortening of the condylar process, as well as an elongation, and widening of the condyle itself. These findings are in agreement with the changes described in patients with rheumatoid arthritis, and juvenile idiopathic arthritis, where destructive changes in the joint area are observed, including bone resorption, cortical, and subcortical erosions, and osteophyte formation [28–30]. Consequently, morphologically, there is an overall flattening of the condyles with a shortening of the condylar process, which, when severe, can lead to lower jaw asymmetry, retrognathia, and joint malfunction [31–33]. Furthermore, our results are consistent with recent work showing jaw joint destruction in mice with TNFα overexpression [20]. In addition, degenerative bone changes play a significant role in RA patients. This can manifest locally, affecting the subchondral, and periarticular bone, as well as in the form of systemic osteoporosis. Recent human pathological studies that used High-Resolution Peripheral Quantitative Computed Tomography (HR-pQCT), and similar parameters to investigate the joint areas of RA patients have yielded similar results. Analyzing the distal radius [34], and the distal tibia or metacarpal heads [35] for example, revealed a significant deterioration in bone parameters.

On a genetic level, this bone loss can be explained, among other factors, by the initial involvement of TNFα in various aspects of bone metabolism. Due to its ability to influence both anabolic osteoblasts (by inducing apoptosis, and inhibiting differentiation factors Runx2, and Osterix), and catabolic osteoclasts (by stimulating differentiation in conjunction with RANKL, and M-CSF), TNFα plays a potent role in bone resorption [5, 36, 37]. Furthermore, studies have shown that immune complexes and autoantibodies play key roles in RA. These findings highlight the need for targeted therapeutic interventions addressing both the inflammatory mediators and the underlying immune pathways involved in arthritis [38].

Therefore, it is important to understand the potential impact of therapy with the relatively new group of TNFα inhibitors on inflammatory bone resorption. However, an osteoprotective effect that goes beyond the expected level achieved by controlling inflammation itself has not yet been conclusively demonstrated [39].

In recent literature, ribosomal S6 kinase *Rsk2* is considered an influential factor in the inflammatory process downstream of TNFα. Studies on arthritis in the knee joint have previously demonstrated that the absence of *Rsk2* results in a significantly earlier onset, and a more severe course of the disease [7]. Furthermore, this kinase, co-activated by ERK, and PDK1, is described as a protective factor against TNFα-induced bone loss, and synovial hyperplasia [7].

To investigate this property in the temporomandibular joint, we examined mice with TNFα overexpression (*hTNFα-tg*), and those with both TNFα overexpression, and simultaneous RSK2 knockout (*hTNFα-tg;Rsk2*^*−/y*^). Although no significant differences were observed between *hTNFα-tg*, and *hTNFα-tg;Rsk2*^*−/y*^ in the analysis of cartilage degeneration, and general geometric morphometry, a closer examination uncovered notable distinctions in ramus length, and synovial membrane thickness. No changes were observed between *Rsk2*^*−/y*^ without TNFα overexpression, and controls, consistent with other studies showing that osteopenia associated with *Rsk2* loss, tends to occur later [4, 7, 26].

Overall, our research results only partially support the protective role of *Rsk2* in the context of inflammatory arthritis in the temporomandibular joint. While uncontrolled synovial membrane proliferation is not yet fully understood, various findings indicate *Rsk2*'s role in this regulatory mechanism [7, 40]. For instance, in the absence of *Rsk2*, Derer et al. 2016 demonstrated increased proliferation of synovial fibroblasts which are considered a driving force in inflammatory arthritis [41]. Expression analyses in the knee joint under *Rsk2* knockout also showed additional increases in TNFα, and various other inflammatory mediators such as IL-1, IL-6, and MMPs [7]. Furthermore, the differing changes observed in craniofacial versus long bones can be attributed to their distinct structural, functional, and developmental characteristics. Factors such as the mechanical loading environment, genetic regulation, and specific responses to pathological conditions play significant roles in shaping how these bone types react to various stimuli.

However, it is important to note that this study has its limitations. One of which is the age of the mice. Despite being young (10 weeks), the mice may not be representative for all age groups, and do not capture the early effects of *Rsk2* on TNFα induced bone resorption in the craniofacial system. Therefore, further research with younger groups is needed to confirm these findings.

Despite the fact the results have proven consistent across different methods, and comparisons the small sample size remains a limitation of our study. Additionally, the study does not explore the underlying mechanisms behind the observed regional differences, which could provide a more complete understanding of the results. Future research addressing these mechanisms would offer a more comprehensive perspective, and help confirm, and extend our findings.

In summary, this study has shown that TNFα overexpression in the temporomandibular joint leads to significant cartilage, and bone loss, and *Rsk2* can exert some protective effects in the inflammatory cascade. While the transgenic mouse model cannot make definitive statements about upstream factors of Tumor necrosis factor, it is extremely helpful in examining the pathomechanism of TNFα, and Rsk2. This understanding of the origin, and control of synovial, and bone changes is ultimately crucial for successful therapy of inflammatory joint diseases. Inhibitors of TNFα have been successfully used in the treatment of rheumatoid, and juvenile arthritis for several years [5]. The insights from our study suggest that other factors within the TNF regulatory pathway, such as *Rsk2*, may also be suitable for better modulating some aspects of the inflammatory process in the future.

## Supplementary Information

Supplementary Material 1.

## Data Availability

Data is provided within the manuscript or supplementary information files. µCT RAW, and Avizo data will be made available on request by the corresponding authors.
