# Supplementary material for: The effect of Rsk2 on TNFα-mediated bone loss in the TMJ and craniofacial skeleton
Source: BMC Oral Health. 2025 Mar 26;25:435. doi: 10.1186/s12903-025-05779-9 (PMC11938757; doi:10.1186/s12903-025-05779-9)
Supplement: Supplementary file 1 — Supplementary Material 1. [file 12903_2025_5779_MOESM1_ESM.docx]

**The effect of Rsk2 on TNFα-mediated bone loss in the TMJ and craniofacial skeleton**

**Authors:** Gina Marie Georgi^1^, Frédéric Bachmann^2^, Julia Luther^3^, Anja Derer^4^, Patrick Heimel^5,6,7^, Stefan Tangl^5,6^, Bärbel Kahl-Nieke^2^, Aaron LeBlanc^8^, Jill Helms^9^, Georg Schett^10,11^, Reinhard Gruber^4^, Michael Amling^3^, Thorsten Schinke^3^, Till Koehne^1^ and Julian Petersen^1§^

**Supplementary Data:**

**Supplementary** **Figure 1** (**A**) Mandible (length) was measured using the distal edge of the articular process to the mesial edge of the alveolar process (**B**) Mandible (height) was measured using the caudal edge of the mandible to the cranial center of the condyle (**C**) Processus condylaris (length) was measured by first drawing a straight line between the two upper and lower points of the articular process. Next, the average distance between these points was measured. (**D**) Condyle (length) was measured by using the distance between the most distal and most mesial point of the condyle (**E**) Condyle (width) was measured using the distance between the lateralmost and medialmost point of the condyle in the frontal plane.


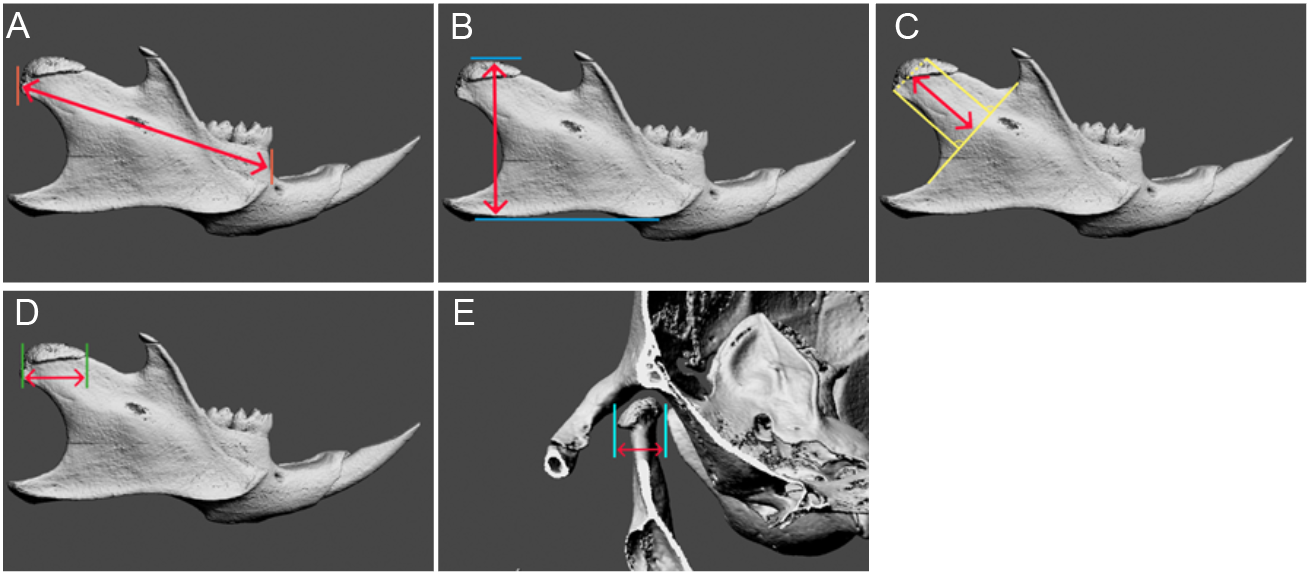


*Detailed Temporomandibular joint measurement using µCT*

µCT Scans were rotated using Fiji (Schindelin, Arganda-Carreras et al. 2012) so the frontal plane is parallel with the X axis of the stack with the nose pointed towards the lower slice numbers and the calvaria pointing upwards. Stacks were then cropped to the mandibular joint and each joint saved as a separate stack. The stack for the right joint was mirrored horizontally so it matches the left joint. The cropped stacks (**Supplementary** **Figure 1A**) were then imported into Defines Developer XD 2.7 (Definiens AG, Munich, Germany).

To reliably separate upper and lower jaws, bone is segmented in two steps. First, voxels above a threshold of 600 mgHA/cm³ with a 3x3x3 median filter are classified as bone (**Supplementary** **Figure 1B**). The objects closest to the upper or lower border of the image above 0.4 mm³ are classified as upper and lower jaw respectively. Voxels above a threshold of 350 mgHA/cm³ are classified with a temporary class. Any objects of the temporary class and floating pieces of bone are added to the lower or upper jaw depending on which is closest to the object (**Supplementary** **Figure 1C**).

Due to the degeneration of the bone, the following process was necessary to find the joint surface. First, the stack was down sampled to 50% and the lower and upper jaw classes were alternatingly coated 11 pixels (330 µm) with different temporary classes to keep them separate followed by shrinking from the outside surface by 4 pixels (120 µm) (**Supplementary** **Figure 1D**). The objects were then shrunk by up to 16 voxels with a surface tension constraint of < 40% in a 5x5x5 voxel volume followed by up to 10 voxels of growing with a surface tension constraint of ≥ 47 % in 5x5x5 voxel volume (**Supplementary** **Figure 1E**). The temporary class is then added the upper and lower jaw classes and synchronized as a void class to the full resolution (**Supplementary** **Figure 1F, G**). The result is, that the gaps inside the bone are now filled with a void class which forms a smooth surface around the bone. However, in some areas, especially when the cortical bone is concave, this void class also exists outside of the bone region. To correct this error, the void classes are shrunk from the outside by 8 voxels (120 µm) which removes any void on the outer surface of the bone. This is followed by growing by 6 voxels (90 µm) which allows the void inside the degenerated joint to grow back toward the surface. A final smoothing run is performed by shrinking the void up to 20 times with a surface tension constraint of < 49% in a 7x7x7 voxel volume followed by growing up to 5 times with a surface tension constraint of ≥ 52 % in 7x7x7 voxel volume (**Supplementary** **Figure 1H**).

To find the joint surface, the stack is rotated around the Z axis by 30° so that the surface of the lower joint is exactly below the surface of the upper joint along the Y axis. The lower joint is coated 1 voxel thick with a temporary class in all directions. The temporary class is then grown by 2 voxels only in the positive direction along the Y axis (toward the top of the image) followed by shrinkage only along the X and Z axis. This creates a connected layer on the surface of the joint with the largest such object representing the joint surface (**Supplementary** **Figure 1I**). This layer is then allowed to grow outward with a surface tension constraint of ≥ 49% in a 9x9x3 voxel volume to further smooth the surface. The resulting surface layer is then limited to the outermost 2 voxels. To find the surface of the upper joint, the joint surface of the lower joint is coated with a temporary class 10 voxels (150 µm) along the Y axis toward the upper joint. This class is then expanded 10 voxels (150 µm) in any direction followed by growing 80 voxels (1.2 mm) along the Y axis. The growing is limited to outside of bone and void classes so the resulting object will touch the joint surface of the upper jaw but not grow into it. The upper jaw is then coated 3 voxels (45 µm) thick into the temporary class, creating the joint surface for the upper jaw. The surface of the upper jaw is limited to the largest continuous surface object (**Supplementary** **Figure 1J**).

To measure the bone erosion, the joint surfaces are coated with a temporary class along the Y axis into the void until it reaches bone or up to 60 voxels (900 µm). Wherever this class hits a voxel of bone it is reclassified and the growth terminates. Bone erosion is then measured as the average distance of those bone voxels to their respective joint surface (**Supplementary** **Figure 1K**). To measure the superficial bone volume fraction, the volume of bone and void within 150 µm from the respective joint surface along the Y axis is measured (**Supplementary** **Figure 1L**).


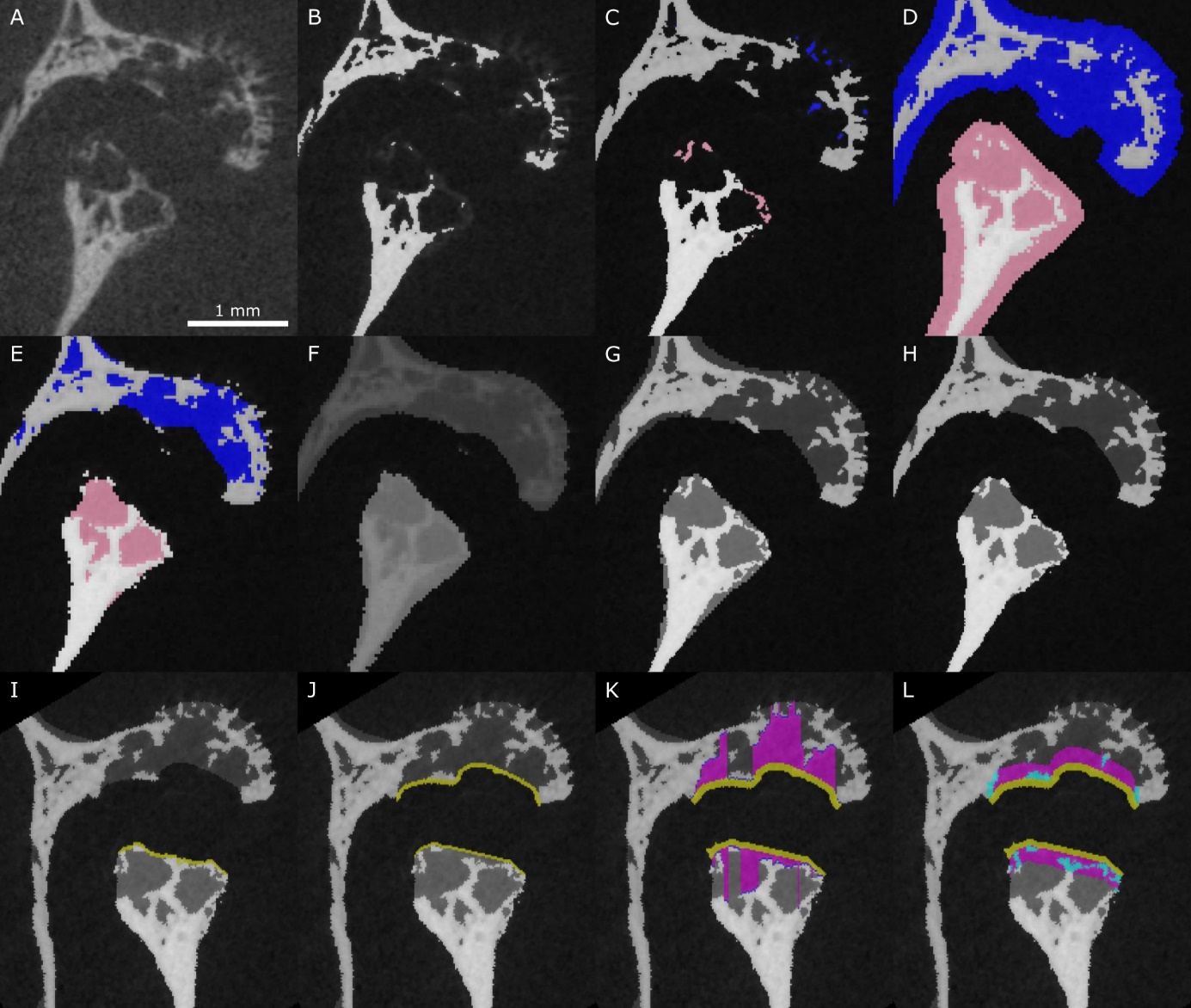


**Supplementary** **Figure 2**: Segmentation process for quantification of the TMJ in Definiens Developer

(**A**) µCT scan cropped to TMJ. (**B**) Segmentation with 600 mgHA/cm³. (**C**) Segmentation with 350 mgHA/cm³ and separation of condyle/fossa. (**D**) Coat bone to find void regions. (**E**) Shrink void candidates back toward bone. (**F**) Smooth surface and reclassify as void. (**G**) Synchronize void to full resolution. (**H**) Correct surface errors and smooth surface. (**I**) Rotate 30° and locate joint surface of condyle. (**J**) Smooth condyle surface and project surface onto fossa. (**K**) Measure bone erosion. (**L**) Measure bone volume fraction.
White represents bone, gray represents void, blue and pink are temporary classes, yellow is the joint surface, magenta is void in the measurement region, cyan is bone in the measurement region.

**C**

|  | **A** |  | **B** |  | **C** |
| --- | --- | --- | --- | --- | --- |
| 11 | Maxilla left | 1 | Os nasale anterior | 1 | Anterior condylar process |
| 12 | Maxilla right | 2 | Nasion | 2 | Posterior condylar process |
| 13 | Orbita anterior left | 3 | Os frontale posterior | 3 | Angulus mandibulae |
| 14 | Orbita anterior right | 4 | Os parietale cranial | 4 | Margo mandibulae posterior |
| 15 | Sutura zygomaticomaxilaris left | 5 | Os parietale cranial posterior | 5 | Margo mandibulae |
| 16 | Orbita lateral left | 6 | Cranium posterior | 6 | Margo mandibulae anterior |
| 17 | Arcus zygomaticus left | 7 | Basis cranii posterior | 7 | Proc. alveolaris |
| 18 | Orbita posterior left | 8 | Basis cranii caudal | 8 | Proc. mandibularis |
| 19 | Arcus zygomaticus post. left | 9 | Basis cranii | 9 | Anterior molar |
| 20 | Sutura zygomaticomaxilaris right | 10 | Basis cranii anterior | 10 | Posterior molar |
| 21 | Orbita lateral right |  |  | 11 | Proc. coronoideus |
| 22 | Arcus zygomaticus right |  |  |  |  |
| 23 | Orbita lateral right |  |  |  |  |
| 24 | Arcus zygomaticus post. right |  |  |  |  |
| 25 | Cranium mediale left |  |  |  |  |
| 26 | Cranium mediale right |  |  |  |  |
| 27 | Cranium lat. superior left |  |  |  |  |
| 28 | Cranium lat. superior right |  |  |  |  |
| 29 | Cranium post. superior left |  |  |  |  |
| 30 | Cranium post. superior right |  |  |  |  |

**Supplementary** **Figure 3** Mouse skull and landmarks used in MorphoJ. (**A** and **B**) Schematic views of the mouse cranium (**A**: superior view; **B**: lateral view) and (**C**) mandible (lateral view).(**C**) Descriptions of the specific landmarks.


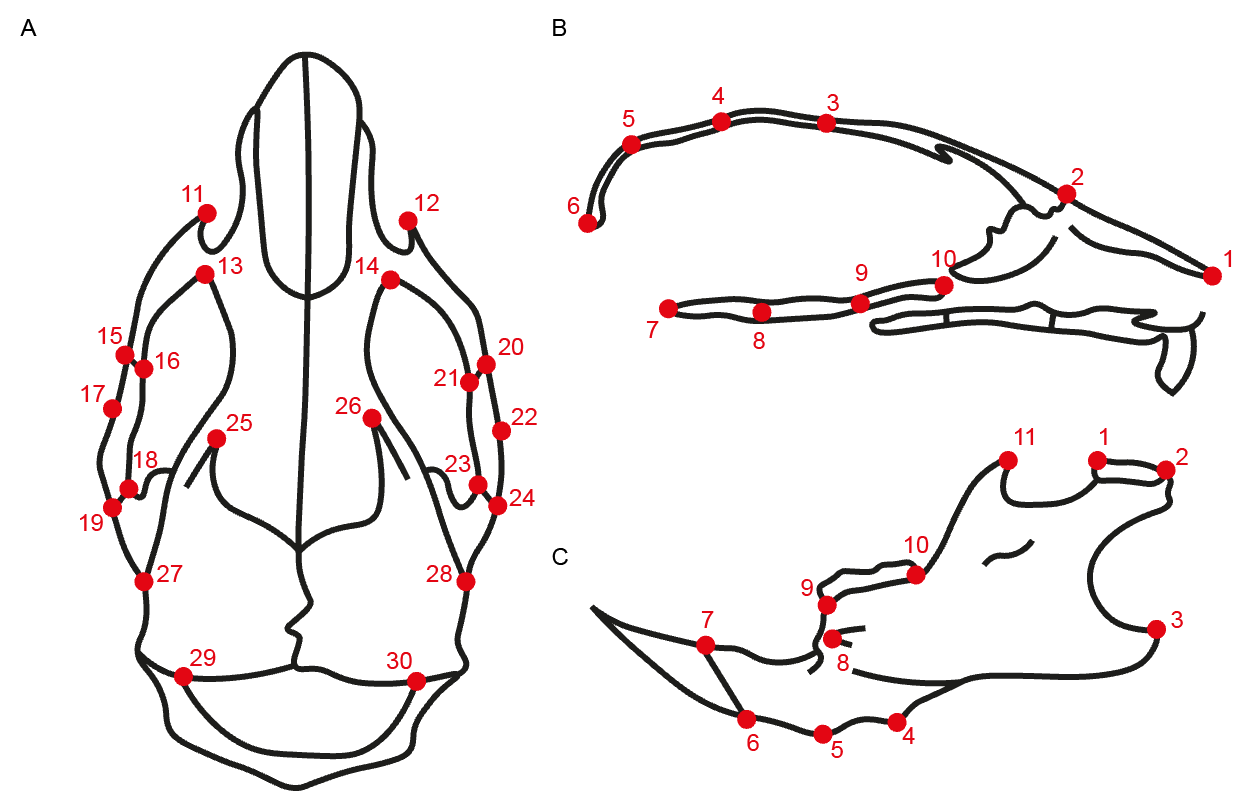


**References**:

Schindelin, J., I. Arganda-Carreras, E. Frise, V. Kaynig, M. Longair, T. Pietzsch, S. Preibisch, C. Rueden, S. Saalfeld, B. Schmid, J. Y. Tinevez, D. J. White, V. Hartenstein, K. Eliceiri, P. Tomancak and A. Cardona (2012). "Fiji: an open-source platform for biological-image analysis." Nat Methods **9**(7): 676-682.
